# Supplementary material for: Fast and robust drift correction for single-molecule localization microscopy
Source: Nat Commun. 2025 Oct 10;16:9031. doi: 10.1038/s41467-025-64085-8 (PMC12514242; doi:10.1038/s41467-025-64085-8)
Supplement: Supplementary file 1 — Supplementary Information [file 41467_2025_64085_MOESM1_ESM.pdf]

SUPPLEMENTAL INFORMATION

Supplementary Figures

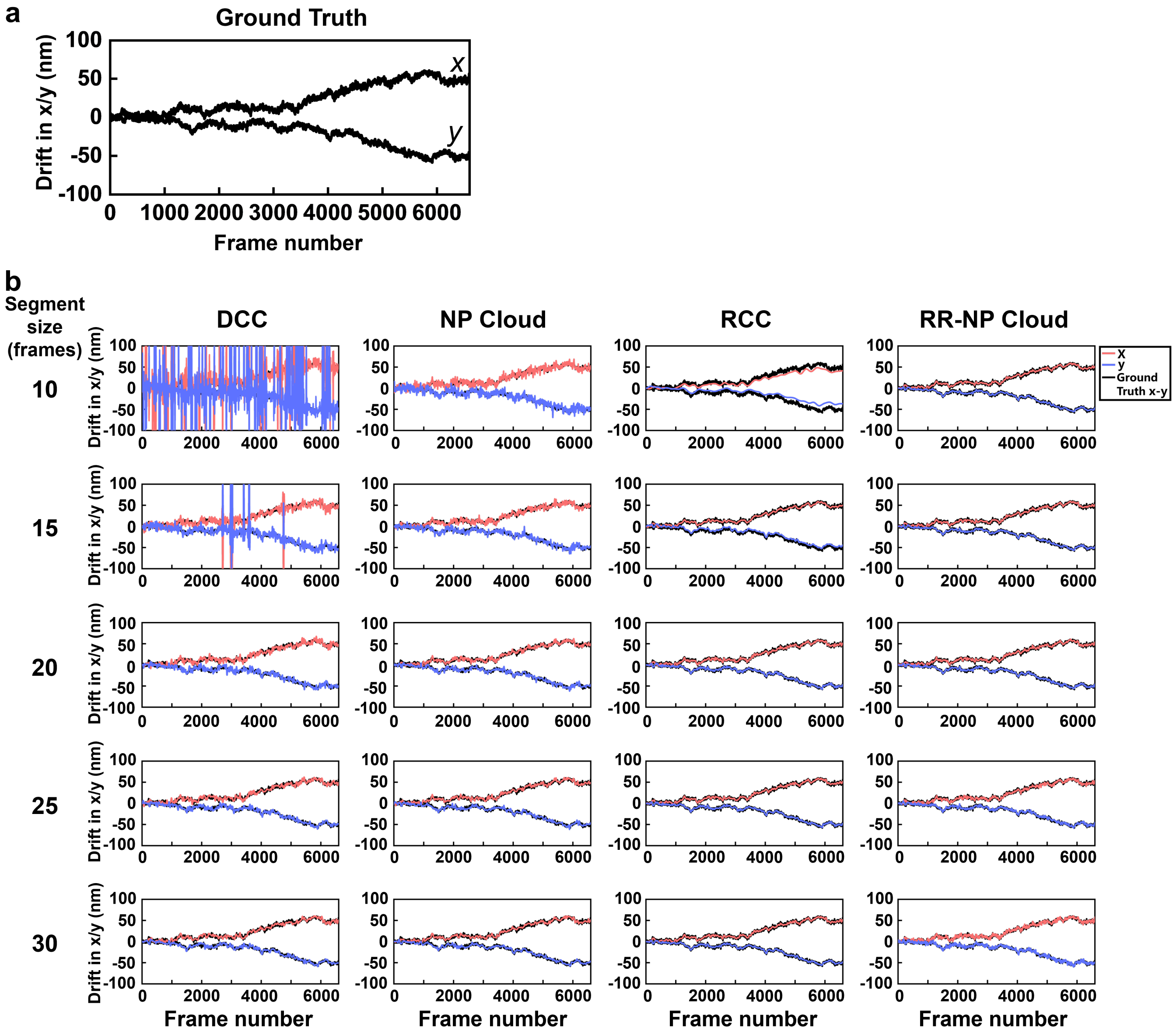

Supplementary Fig. 1. Comparing the drift-correction results of NP-Cloud and RR-NP Cloud with traditional approaches of DCC and RCC, for the simulated short SMLM dataset of 6,600 frames with sparse features of 10 clusters/μm^2. (a) Ground truths of the drift curves in *x* and *y*, which were obtained experimentally on an SMLM setup using fluorescent beads and then applied to the simulated data to generate frame-dependent drifts of single-molecule locations. (b) Drift curves calculated from the simulated SMLM data using DCC, NP-Cloud, RCC, and RR-NP Cloud, for the different segment sizes of 10, 15, 20, 25, and 30 frames/segment, plotted for *x* (red) and *y* (blue) versus the ground truth (black).

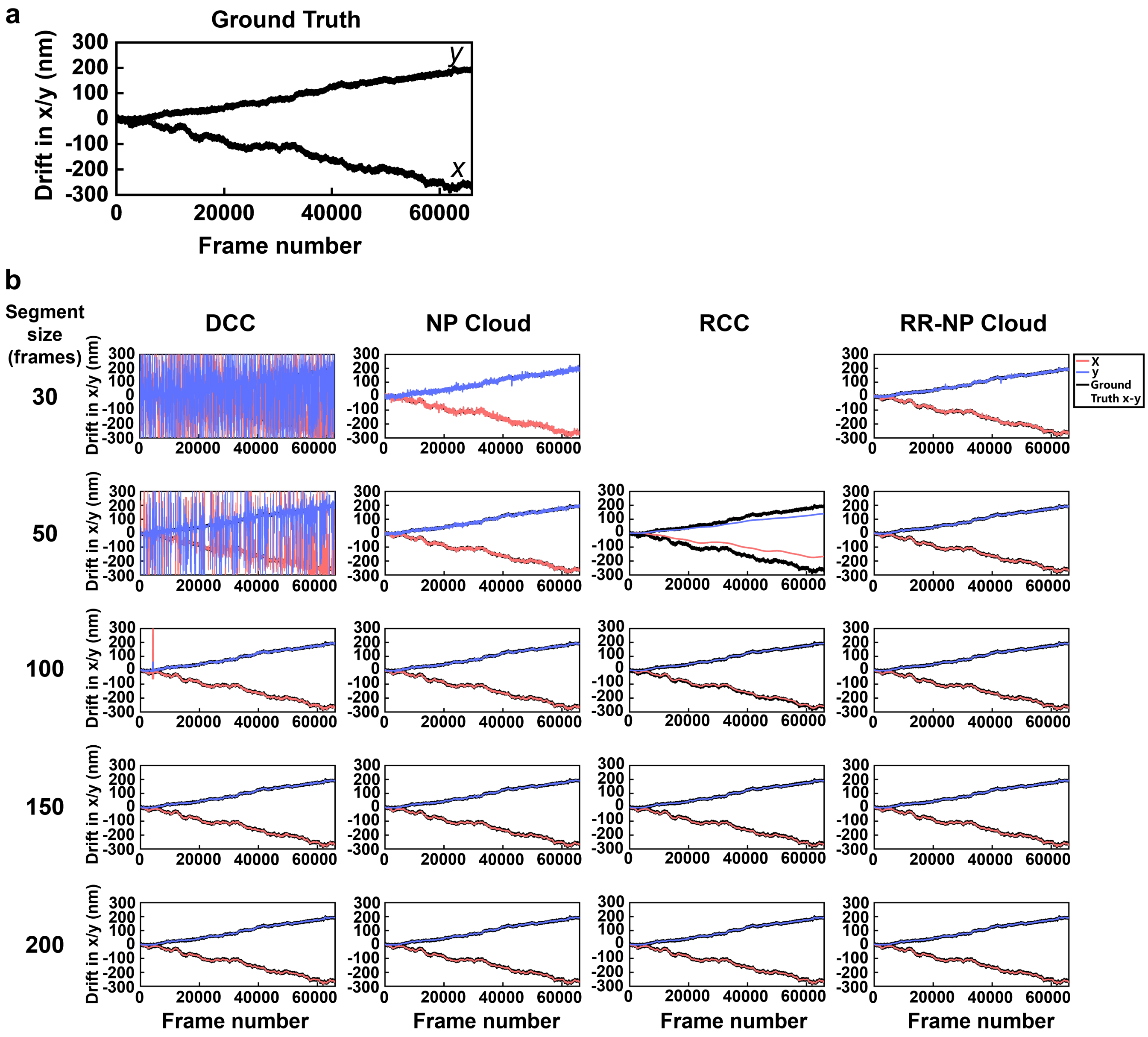

Supplementary Fig. 2. Comparing the drift-correction results of NP-Cloud and RR-NP Cloud with traditional approaches of DCC and RCC, for the simulated long SMLM dataset of 66,000 frames with denser features of 100 clusters/μm^2. (a) Ground truths of the drift curves in *x* and *y*, which were obtained experimentally on an SMLM setup using fluorescent beads and then applied to the simulated data to generate frame-dependent drifts of single-molecule locations. (b) Drift curves calculated from the simulated SMLM data using DCC, NP-Cloud, RCC, and RR-NP Cloud, for the different segment sizes of 30, 50, 100, 150, and 200 frames/segment, plotted for *x* (red) and *y* (blue) versus the ground truth (black). Note: For the segment size of 30 frames/segment, RCC was unable to complete after many days, and so is omitted.

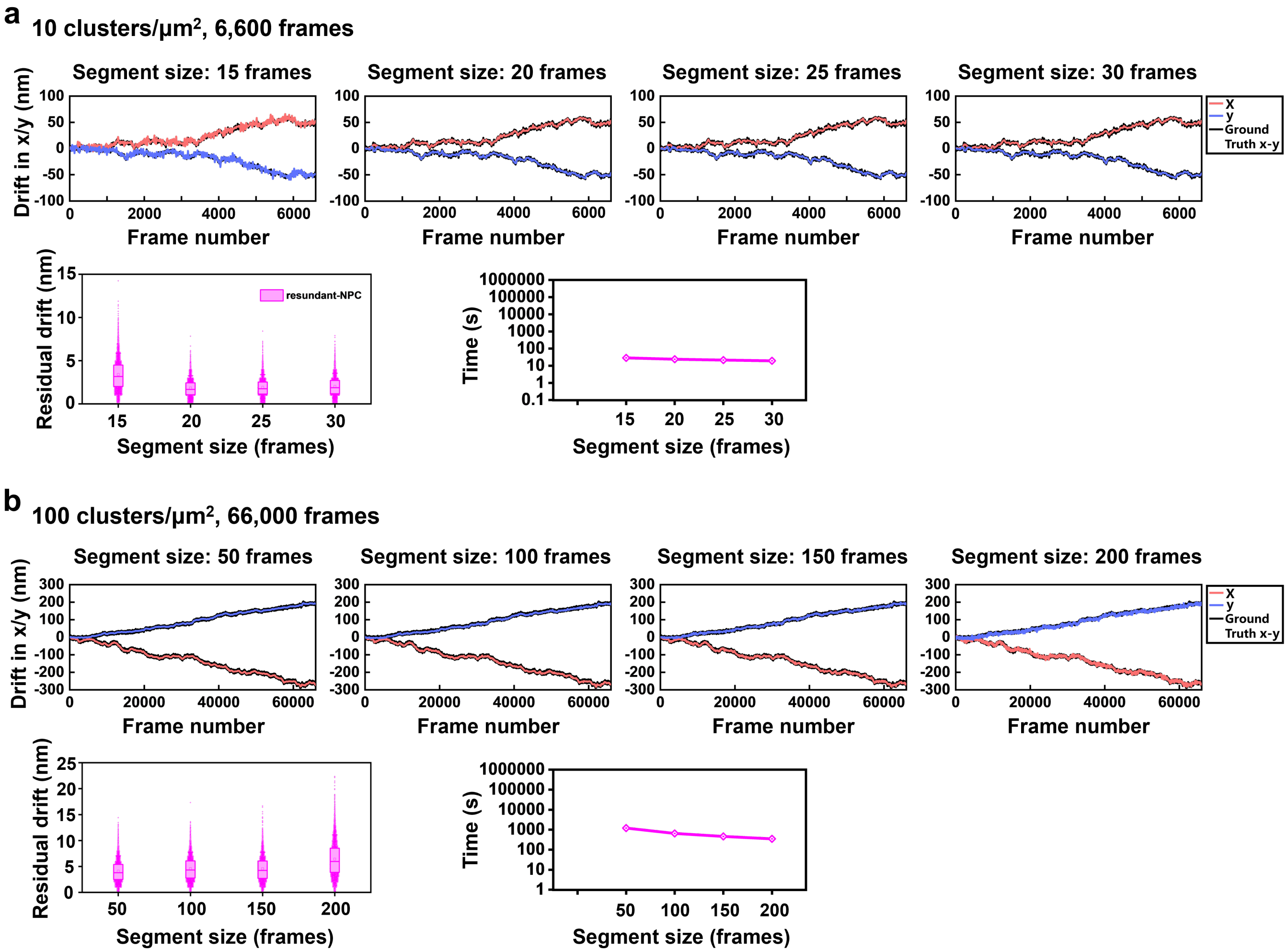

Supplementary Fig. 3. Drift correction of the simulated SMLM datasets with redundant NP-Cloud. Here, similar to the treatment in RCC, we used NP-Cloud to cross-examine the relative shifts between all segments, and then solved a matrix to find a drift-correction solution that minimizes the residual drifts between all segments. (a) and (b) show results with the simulated short and long SMLM datasets of 6,600 and 66,000 frames, respectively. Calculated drift curves and resultant residual drift versus the ground truth, as well as the time spent, were plotted for varying segment sizes.

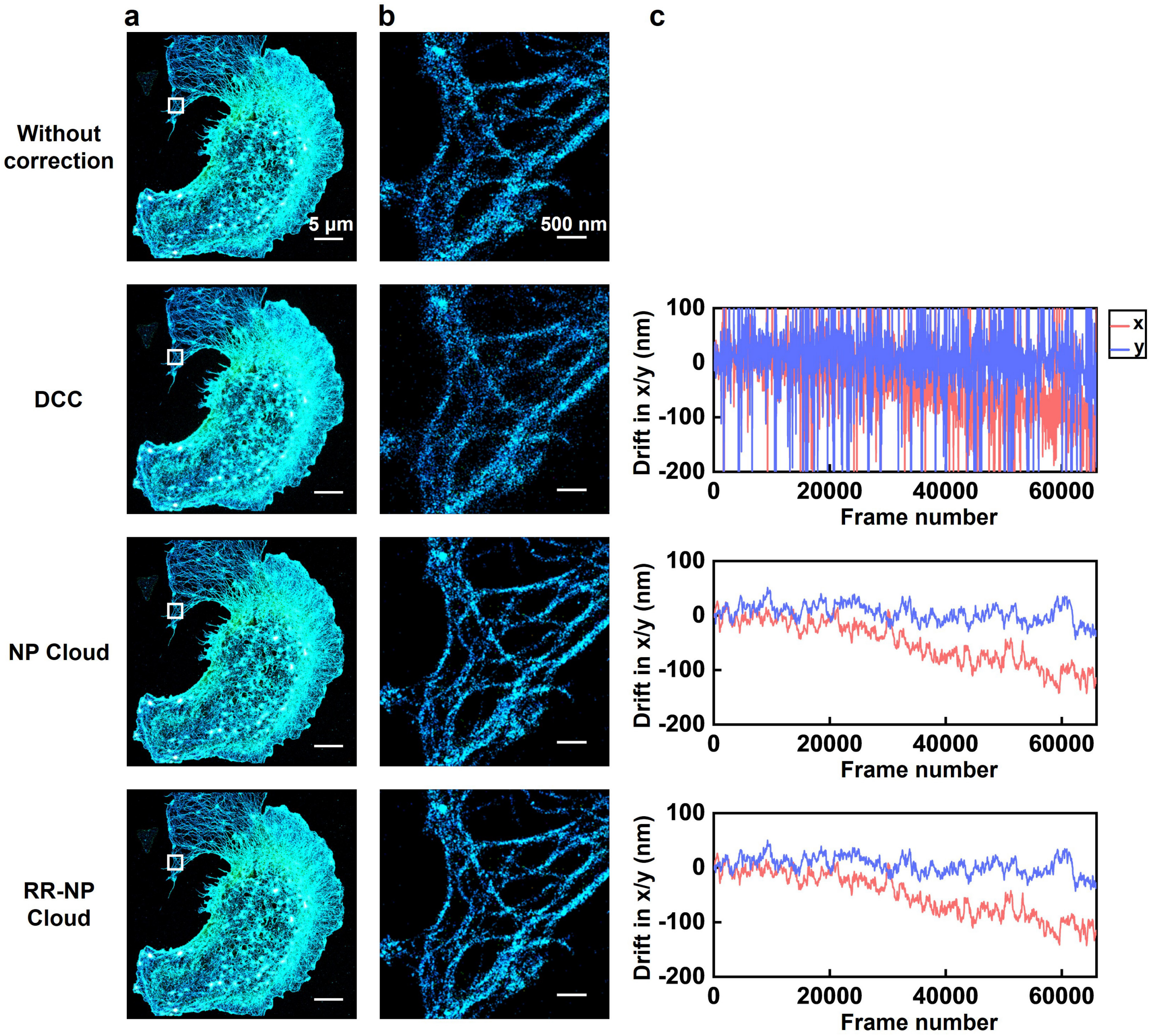

Supplementary Fig. 4. Additional example results on experimental data: STORM of phalloidin AF647-labeled actin filaments in fixed macrophages. (a) Full frame images without and with drift corrections based on DCC, NP-Cloud, and RR-NP Cloud, at the same segment size of 50 frames/segment. (b) Zoom-ins for the boxes in (a). (c) Drifts in x (red) and y (blue) calculated by the different methods.

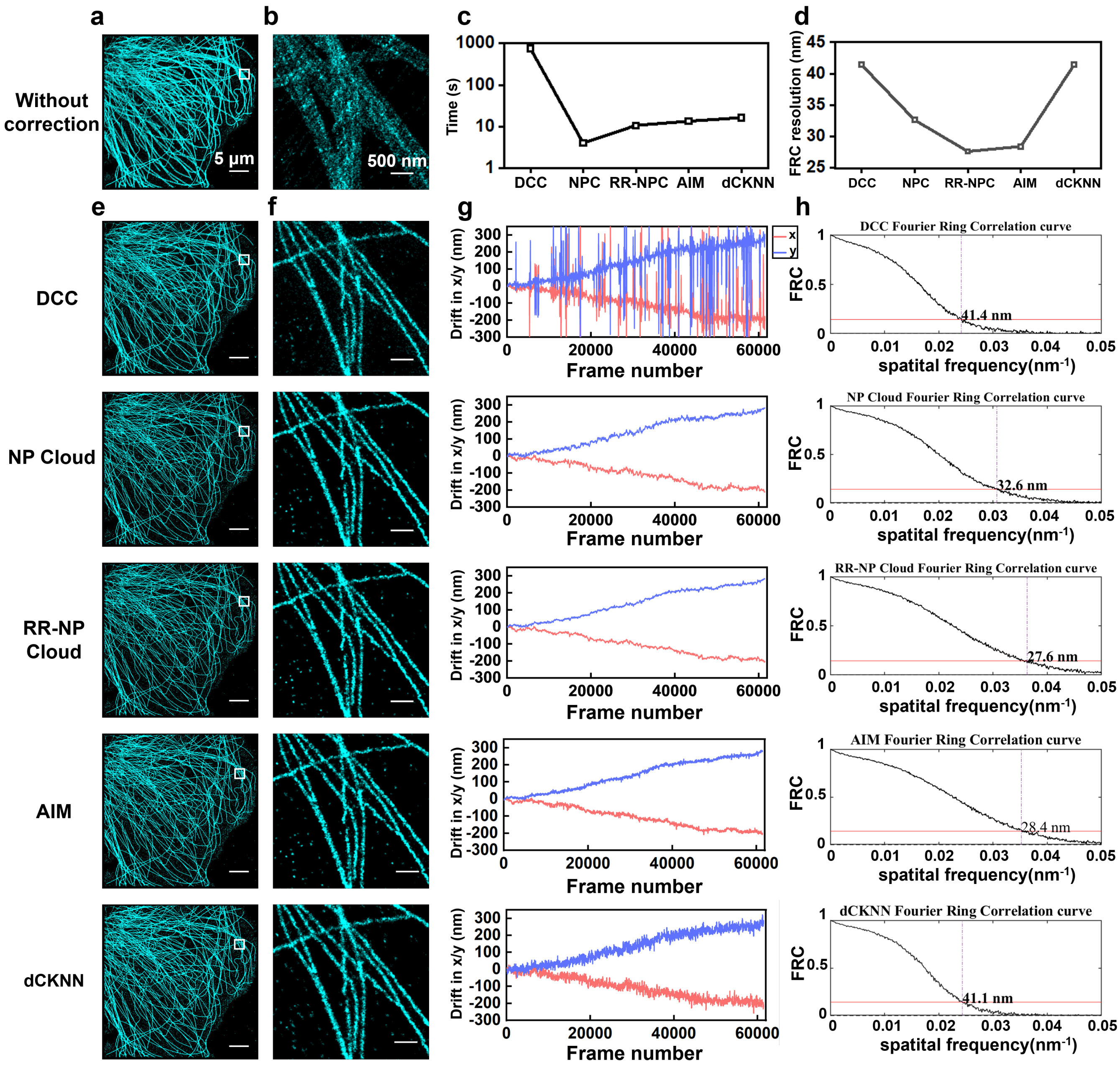

Supplementary Fig. 5. Additional example results on experimental data: STORM of AF647-immunolabeled microtubules (tubulin) in fixed COS-7 cells. (a) Full frame image before drift correction. (b) Zoom-ins of the box in (a). (c,d) Comparison of the time spent (c) and the Fourier Ring Correlation (FRC) resolutions (d) of the drift-corrected results through DCC, NP-Cloud, RR-NP Cloud, AIM, and driftCorrectKNN at the same segment size of 50 frames/segment. (e,f) Drift-corrected images from the different methods. (g) Drifts in x (red) and y (blue) calculated by the different methods. (h) FRC analysis of the drift-corrected data from the different methods, with resolution values marked in the plots. Note that we also attempted RCC and mean-shift methods under the same conditions, but they did not complete after >7 days.

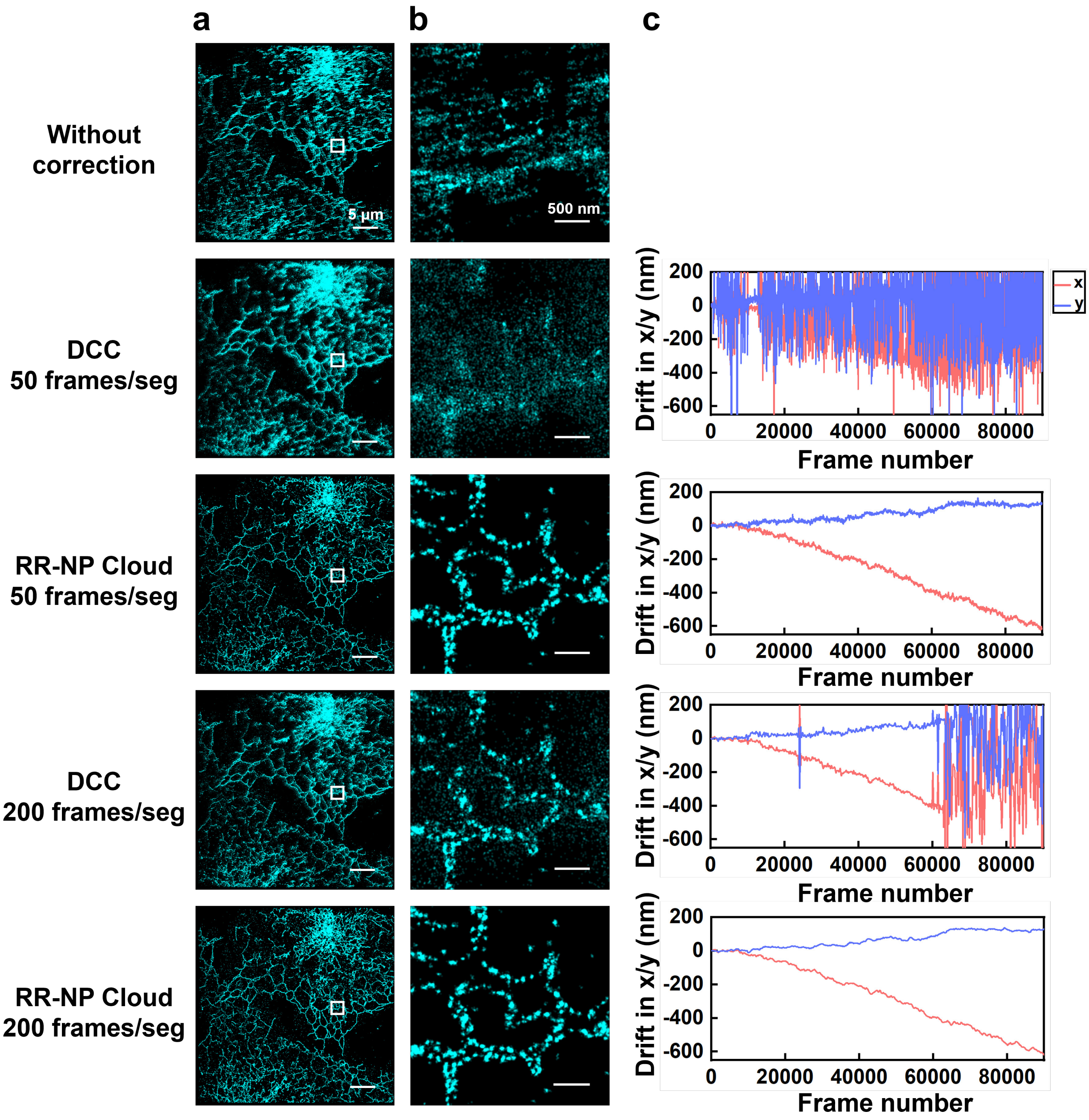

Supplementary Fig. 6. Additional example results on experimental data: STORM of AF647-immunolabeled reticulon Rtn4 (Nogo), an endoplasmic reticulum sheet-edge/tubule protein, in fixed COS-7 cells. (a) Full frame images without and with drift corrections based on DCC and RR-NP Cloud at two different segment sizes of 50 and 200 frames/segment. (b) Zoom-ins for the boxes in (a). (c) Corresponding drifts in x (red) and y (blue) calculated by the different methods. RR-NP Cloud worked well for both segment sizes, while DCC failed at 50 frames/segment and is still not robust at 200 frames/segment, especially for later parts of the data, when the count of localizations in each frame dropped due to photobleaching, a common scenario in SMLM.

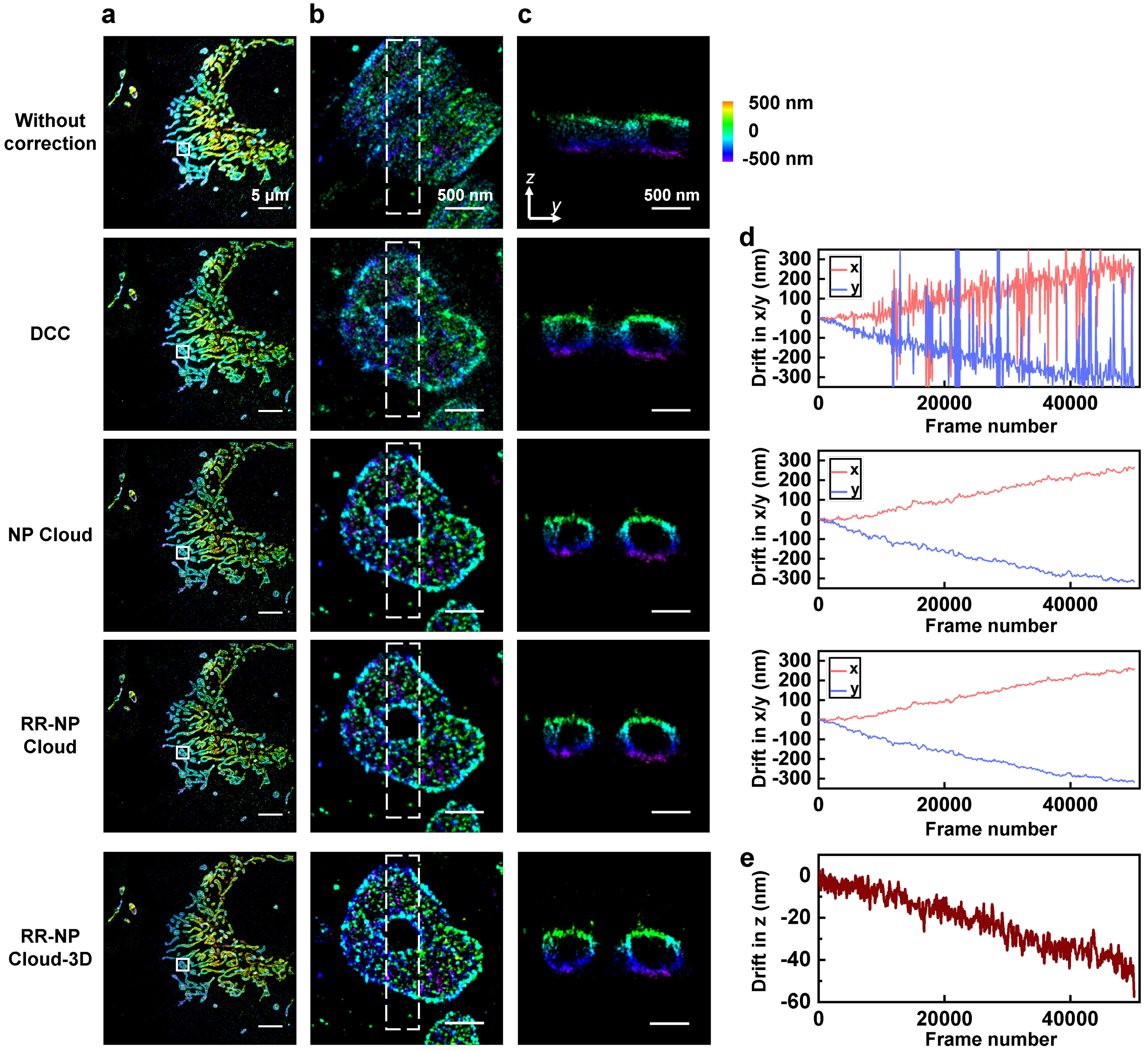

Supplementary Fig. 7. Example results on 3D-STORM data of AF647-immunolabeled mitochondrial outer membrane (TOM20) in fixed COS-7 cells. Here, a focal lock held the focus during data acquisition. (a) Full frame images without and with drift corrections based on DCC, NP-Cloud, RR-NP Cloud, and RR-NP Cloud-3D, at the same segment size of 100 frames/segment. Color presents depth (*z*). (b) Zoom-ins for the boxes in (a). (c) Virtual vertical (*yz*) cross sections along the boxes marked in (b), showing the hollow cross-section of the mitochondrial outer membrane. (d) Drifts in *x* (red) and *y* (blue) calculated by the different methods. (e) Drift in *z* calculated by RR-NP Cloud-3D.

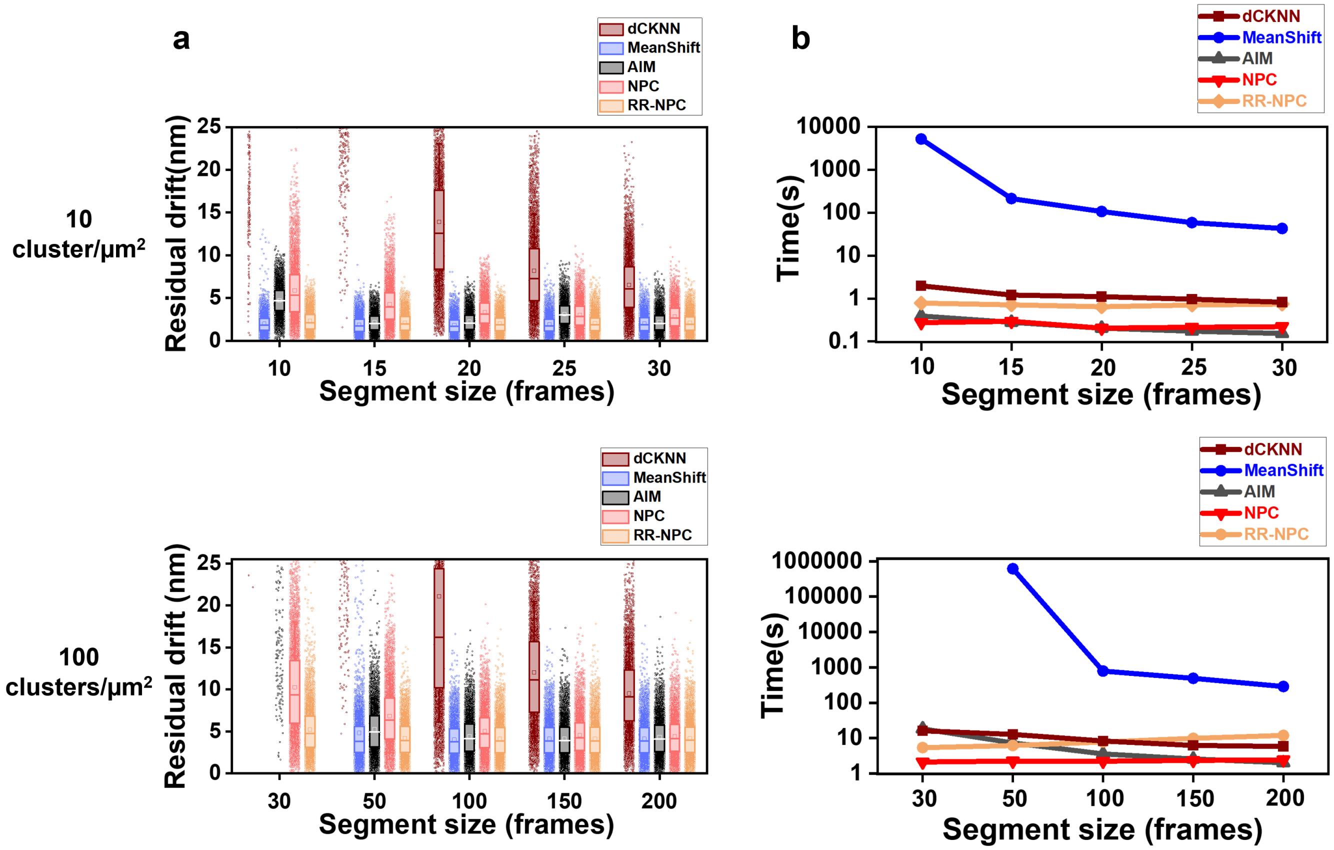

Supplementary Fig. 8. Comparison of drift-correction results of NP-Cloud and RR-NP Cloud with driftCorrectKNN, mean shift, and AIM. (a) Distributions of residual drift in each frame, calculated as the absolute distance between the estimated drift and the ground truth at different segment sizes, for the simulated SMLM dataset of 6,600 frames with relatively sparse features of 10 clusters/μm^2 (top) and for the simulated SMLM dataset of 66,000 frames with denser features of 100 clusters/μm^2 (bottom). (b) Corresponding time spent under the different conditions. Note: For the segment size of 30 frames/segment of the denser SMLM dataset, the mean-shift method was unable to complete after >15 days, and so it is omitted in the plots.

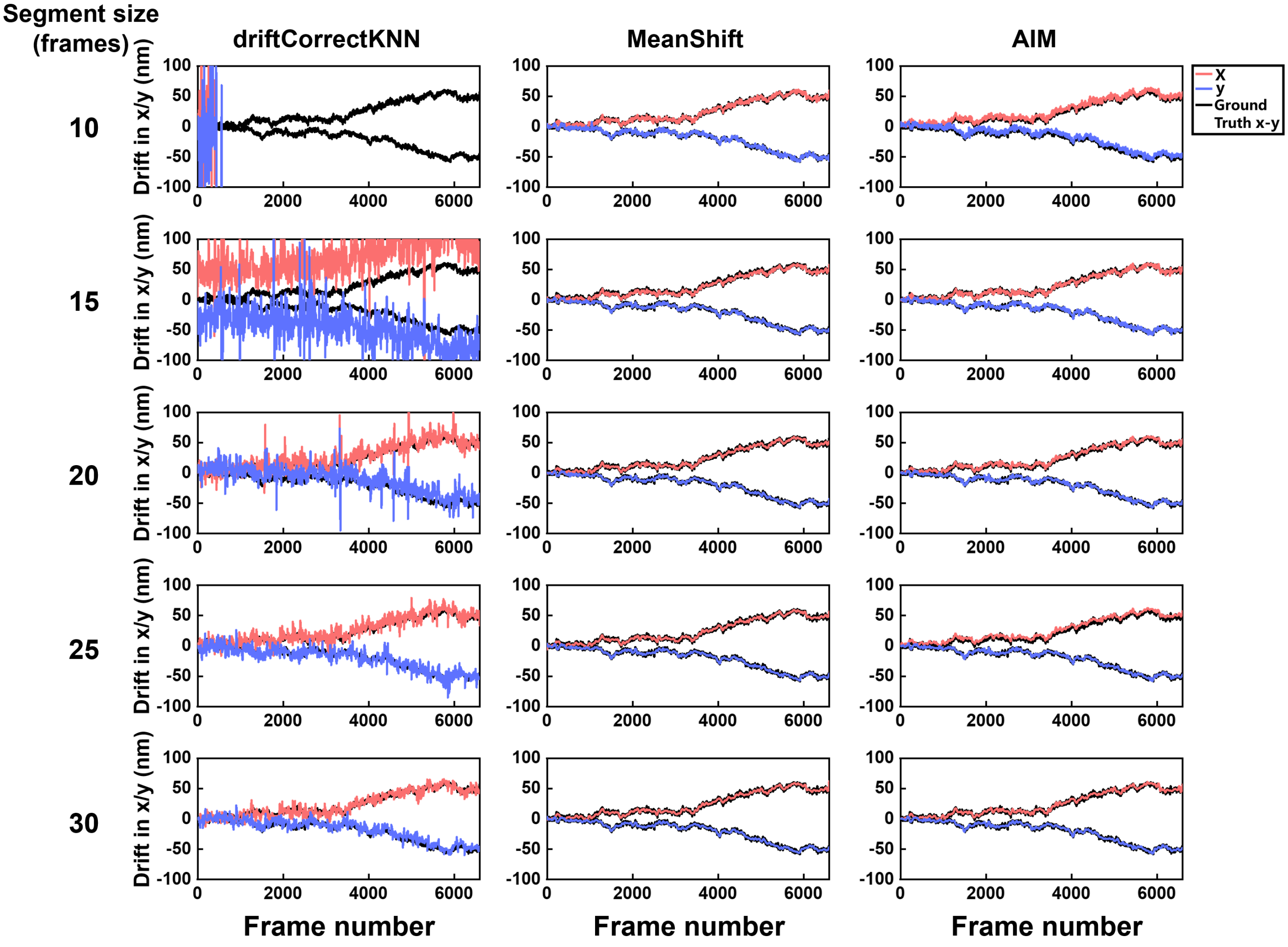

Supplementary Fig. 9. Drift curves calculated from the simulated SMLM dataset of 6,600 frames with relatively sparse features of 10 clusters/μm^2 using driftCorrectKNN, mean shift, and AIM, for the different segment sizes of 10, 15, 20, 25, and 30 frames/segment, plotted for *x* (red) and *y* (blue) versus the ground truth (black).

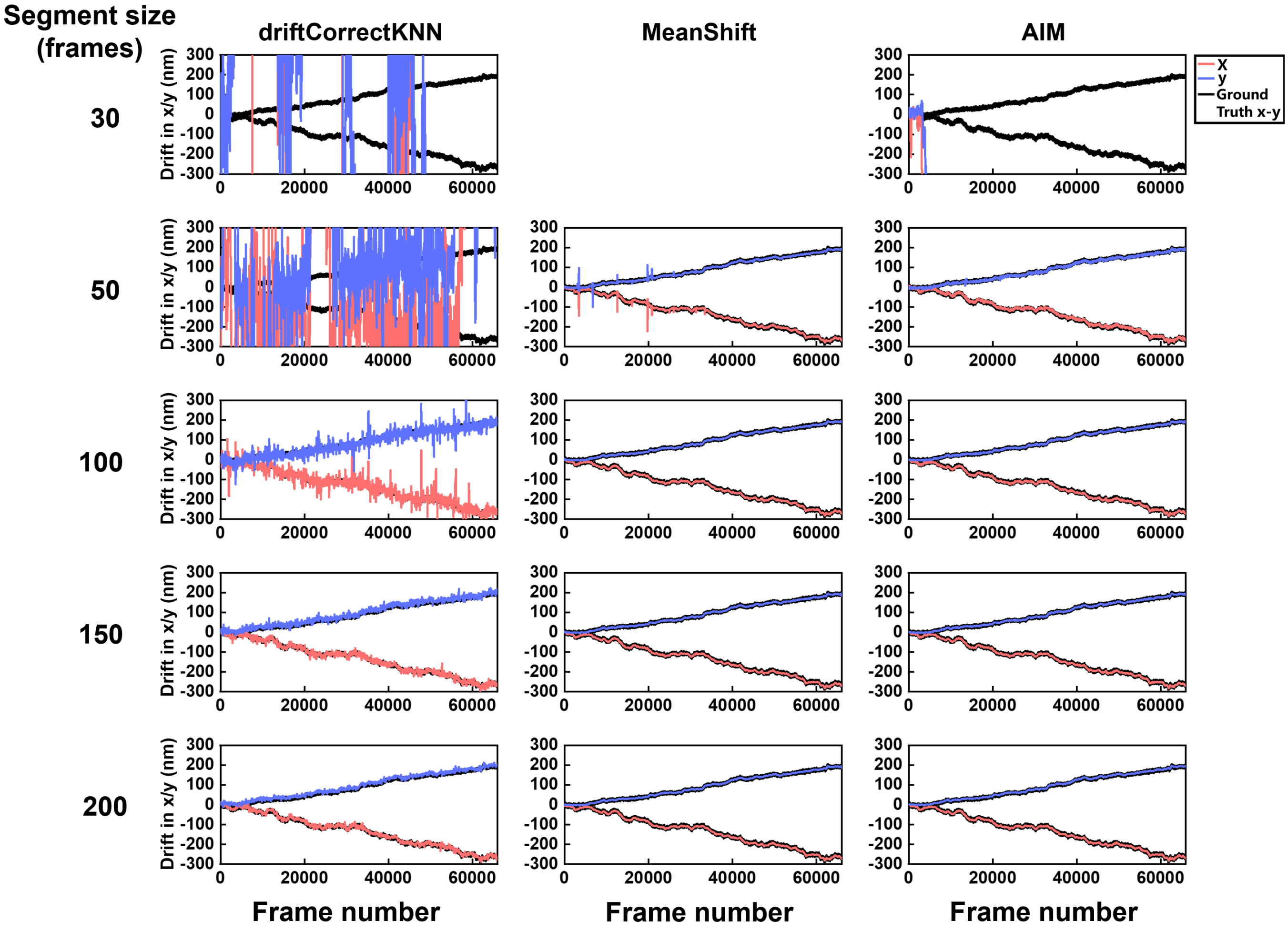

Supplementary Fig. 10. Drift curves calculated from the simulated SMLM dataset of 66,000 frames with denser features of 100 clusters/μm^2 using driftCorrectKNN, mean shift, and AIM, for the different segment sizes of 30, 50, 100, 150, and 200 frames/segment, plotted for x (red) and y (blue) versus the ground truth (black). Note: For the segment size of 30 frames/segment, the mean-shift method was unable to complete after >15 days, and so it is omitted in the plots.

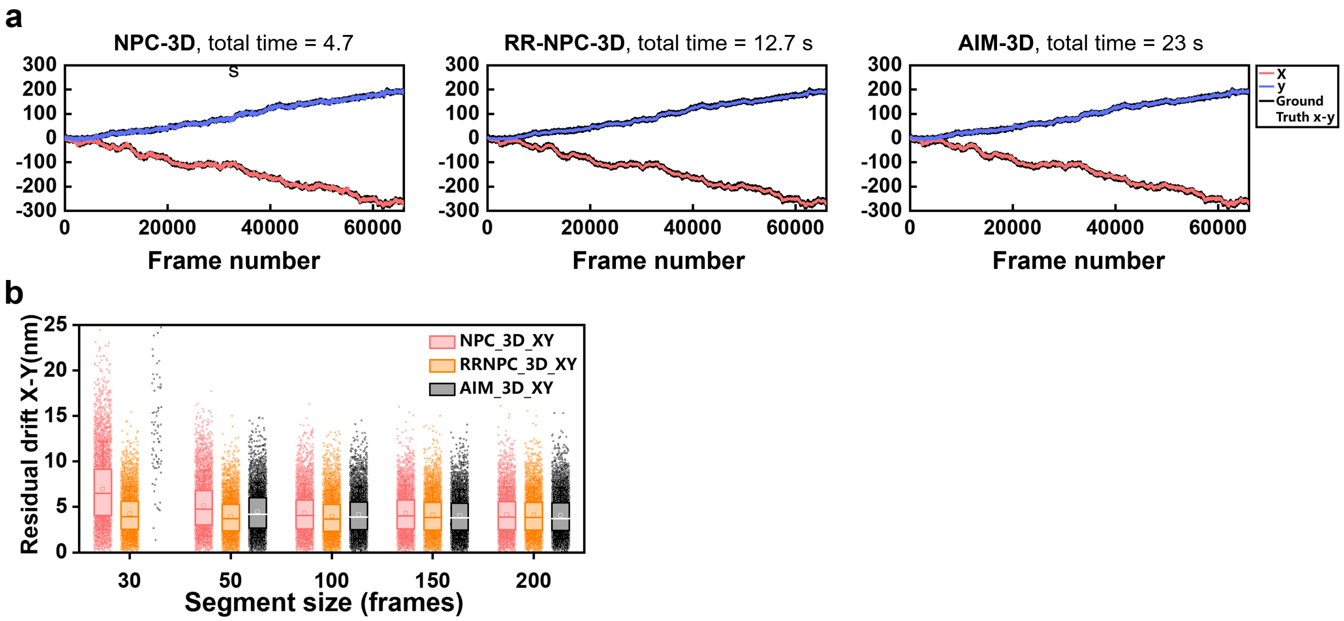

Supplementary Fig. 11. In-pane (*xy*) drift curves obtained from the 3D drift-correction calculations. (a) The in-pane (*xy*) drift curves concurrently obtained with the *z* drift curves shown in Figure 4b for the three 3D drift-correction approaches (NP-Cloud-3D, RR-NP Cloud-3D, and AIM-3D), plotted for *x* (red) and *y* (blue) versus the ground truth (black). (b) Distributions of residual in-plane (*xy*) drift of 5,000 randomly sampled frames from the drift-correction results, calculated as the in-plane (*xy*) distance between the estimated drift and the ground truth, for the three methods at different segment sizes. Note that AIM-3D failed for the 30 frames/segment condition and did not generate meaningful drift correction.

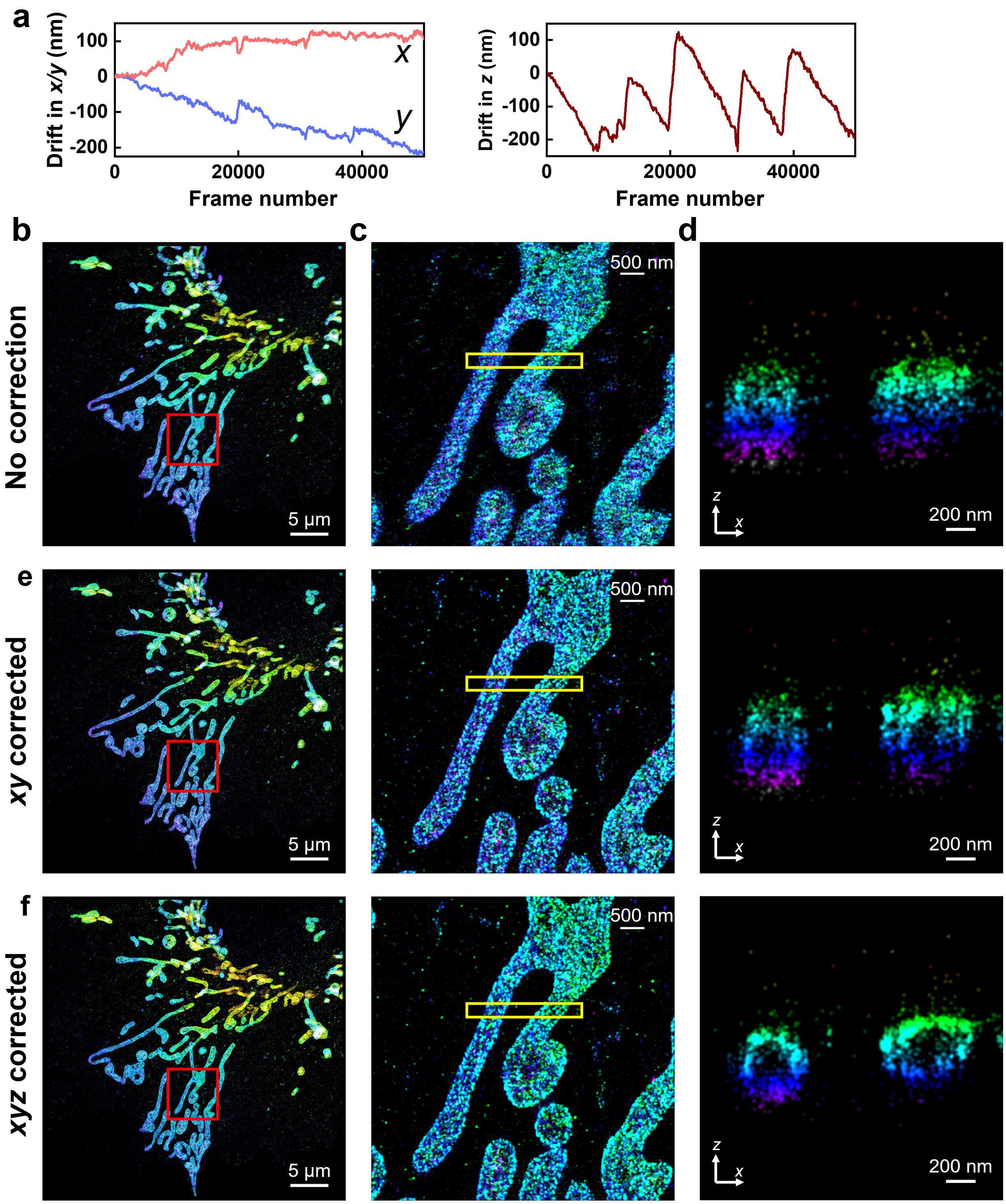

Supplementary Fig. 12. Additional images related to the 3D-STORM data shown in Figure 4hi. When collecting this dataset, the focus lock was disabled, and the sample was allowed to continuously drift in *z* by ~200 nm each time before the focus was manually adjusted to bring the sample back. (a) RR-NP Cloud-3D calculated *xy* (left) and *z* (right) drift curves. (b) Full frame 3D-STORM image without drift correction. Color presents depth (*z*). (c) Zoom-in of the red box in (b). (d) A virtual vertical (*xz*) cross section along the yellow boxed region in (c). (e) Similar to (b-d), but after in-plane (*xy*) RR-NP Cloud drift correction. (f) Similar to (b-d), but after RR-NP Cloud-3D drift correction.
